# A Genomic Approach to Investigating Ocular Surface Microorganisms: Monitoring Core Microbiota on Eyelid Margin with a Dot hybridization Assay

**DOI:** 10.3390/ijms21218299

**Published:** 2020-11-05

**Authors:** Ming-Tse Kuo, Tsai-Ling Chao, Shu-Fang Kuo, Chun-Chih Chien, Alexander Chen, Yu-Hsuan Lai, Yu-Ting Huang

**Affiliations:** 1 Department of Ophthalmology, Kaohsiung Chang Gung Memorial Hospital and Chang Gung University College of Medicine, Kaohsiung 83301, Taiwan; b9902055@cgmh.org.tw (A.C.); hurray@cgmh.org.tw (Y.-H.L.); r06100813@cgmh.org.tw (Y.-T.H.); 2 Department of Laboratory Medicine, Kaohsiung Chang Gung Memorial Hospital and Chang Gung University College of Medicine, Kaohsiung 83301, Taiwan; tsaeling@cgmh.org.tw (T.-L.C.); ivykuo@cgmh.org.tw (S.-F.K.); jessica0307@cgmh.org.tw (C.-C.C.); 3 Department of Medical Biotechnology and Laboratory Sciences, College of Medicine, Chang Gung University, Taoyuan 333323, Taiwan

**Keywords:** ocular surface, microbiota, antibiotic resistance, cataract, endophthalmitis

## Abstract

A sound ocular surface microbiota has been recognized as a part of ocular surface health following a growing body of evidence from next-generation sequencing technique and metagenomic analysis. However, even from the perspective of contemporary precision medicine, it is difficult to directly apply these new technologies to clinical practice. Therefore, we proposed a model based on dot hybridization assay (DHA) to bridge conventional culture with a metagenomic approach in investigating and monitoring ocular surface microbiota. Endophthalmitis, mostly caused by bacterial infection, is the most severe complication of many intraocular surgeries, such as cataract surgery. Hazardous microorganisms hiding and proliferating in the ocular surface microbiota not only increase the risk of endophthalmitis but also jeopardize the effectiveness of the preoperative aseptic procedure and postoperative topical antibiotics. The DHA model enables the simultaneous assessment of bacterial bioburden, detection of target pathogens and microorganisms, and surveillance of methicillin/oxacillin resistance gene *mecA* in the ocular surface microbiota. This assay revealed heavier bacterial bioburden in men, compatible with a higher risk of endophthalmitis in male patients who underwent cataract surgery. No occurrence of endophthalmitis for these patients was compatible with non-hazardous microorganisms identified by specific dots for target pathogens. Moreover, the mecA dot detected oxacillin-resistant strains, of which culture failed to isolate. Therefore, the DHA model could provide an alternative genomic approach to investigate and monitor ocular surface microorganisms in clinical practice nowadays.

## 1. Introduction

The microbiome has been linked to cancer, obesity, asthma, rheumatoid arthritis, and diabetes, illustrating the significance of gut microbiota in health and illness [1,2,3,4]. It is now understood that the human body harbors as many microbial species as human cells [5]. Whether the ocular surface, like other mucous membrane surfaces, has a resident microbiota has been debated over many years [6]. Unlike any other body sites, the heathy conjunctiva, lid margins, and tears have remarkably fewer microbial species than other mucosa sites, such as oral mucosa surface [7]. Blinking and tears with natural killing components for microbes may cause much fewer microorganisms on the cornea and conjunctiva than other body sites. The specific defense mechanisms of the ocular surface may lead to a more unstable and rapidly refreshed microbiota on cornea and conjunctiva than lid margin. According to a recent review [8], the homeostasis of ocular surface microbiota can be easily altered by dry eye disease, antibiotic usage, infections, and contact lens usage. The disruption of the normal eye microbiota can play a significant role in the pathogenesis of ophthalmic diseases, and the alteration of the microbiota of other body sites can also facilitate the development of ophthalmic pathologies. Microbiota on the ocular surface may carry drug-resistant bacterial strains, and antimicrobial peptides were consequently investigated to resolve the problem of antibiotic resistance [9].

Culture-based studies showed the most frequent species identified on the ocular surface is coagulase-negative staphylococci (CoNS), which include *Staphylococcus epidermidis* (*S. epidermidis*) at most [10]; The Gram-negative bacteria, including *Pseudomonas aeruginosa* (*P. aeruginosa*), *Enterobacter* sp., *Escherichia coli*, *Proteus* sp., and *Acinetobacter* sp., are less frequently detected on the healthy ocular surface [9,10,11]. Graham et al. [12] found most of the bacteria identified by culture were CoNS, whereas molecular methods demonstrated a considerable number of additional bacteria. Atypical ocular surface bacteria, including *Rhodococcus erythropolis*, *Klebsiella oxytoca*, and *Erwinia* sp., were also identified in the normal ocular surface. Dong et al. applied pyrosequencing [13] and bioinformatics analysis and found that 12 genera—*Pseudomonas*, *Propionibacterium*, *Bradyrhizobium*, *Corynebacterium*, *Acinetobacter*, *Brevundimonas*, *Staphylococci*, *Aquabacterium*, *Sphingomonas*, *Streptococcus*, *Streptophyta*, and *Methylobacterium*—were ubiquitous among the analyzed cohort. Shin et al. used a similar metagenomics technique and found a higher abundance of *Haemophilus*, *Streptococcus*, *Staphylococcus*, and *Corynebacterium* in the conjunctiva than the skin of the eye, supporting the concept of the ocular commensal signature [14].

However, some fastidious bacteria may not be detected by culture, while some nonviable bacteria were falsely detected by Sanger sequencing since the mixed bacterial DNAs downgraded the quality of amplicons, causing a failed sequencing. Ocular surface microbiomes cannot be correlated well with Sanger sequence and pyrosequencing, too [13,14]. Although metagenomics analysis implied an abundant ocular microbial community, the discrepant results among studies that applied pyrosequencing may be due to environmental bacterial contaminations, sampling differences, biases arising from microbial database diversity, and data pre-processing algorithms of bioinformatics [15,16]. The noise from contaminants to a small community of ocular microorganisms may easily deviate the signal from the ocular microbiome [6,17]. Considering the limitations from the above modalities in understanding microbiota on personal ocular surface, the DNA dot hybridization assay (DHA) may provide a more rational way by detecting target microorganisms [18,19], quantifying microbial burden [20,21], and surveilling antibiotic resistance [22] if the core ocular surface microbiota (COSM) is defined.

Therefore, we proposed a postulated COSM, in which target microorganisms are more frequently recovered or recognized as pathogens or flora from the ocular surface by culture and Sanger sequencing methods. A specially-designed DHA, targeting the predefined COSM with simultaneous assessment for microbial burden and surveillance for antibiotic resistance, was developed for evaluating the COSM of the lid margin. Cataract surgery is the most common ocular surgery, of which endophthalmitis is the most serious complication of this surgery and a nightmare for cataract surgeons. Therefore, this study selected patients who underwent cataract surgeries in order to explore the microorganism profile in the COSM using the DHA model.

## 2. Results

### 2.1. A DHA Model for Postulated COSM and Pre-Test Analysis

**Figure 1:**
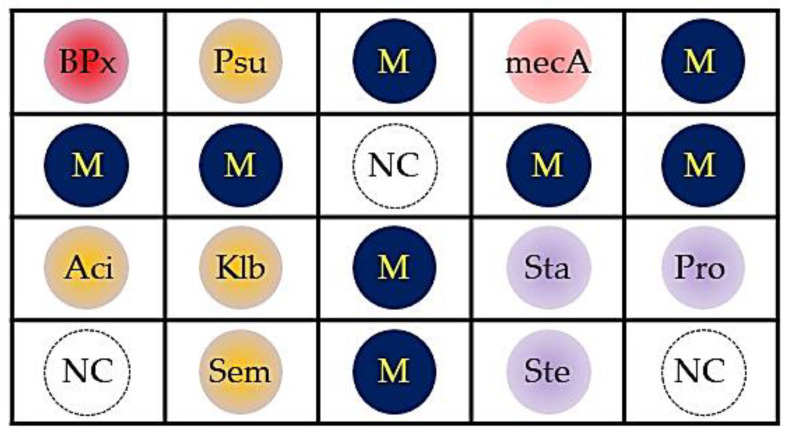
A schematic dot hybridization model for detecting a putative core ocular surface microbiota. The postulated core ocular surface microbiota (detecting dot) included *Pseudomonas aeruginosa* (Psu), *Acinetobacter baumannii* (Aci), *Klebsiella pneumoniae* (Klb), *Serratia marcescens* (Sem), *Staphylococcus aureus* (Sta), *Staphylococcus epidermidis* (Ste), and *Propionibacterium acnes* (Pro). In addition, there was one dot for determining microbial burden (BPx) and another for surveilling antibiotic resistance of microorganism (mecA). M = marker; NC = negative control (tracking dye).

**Figure 2:**
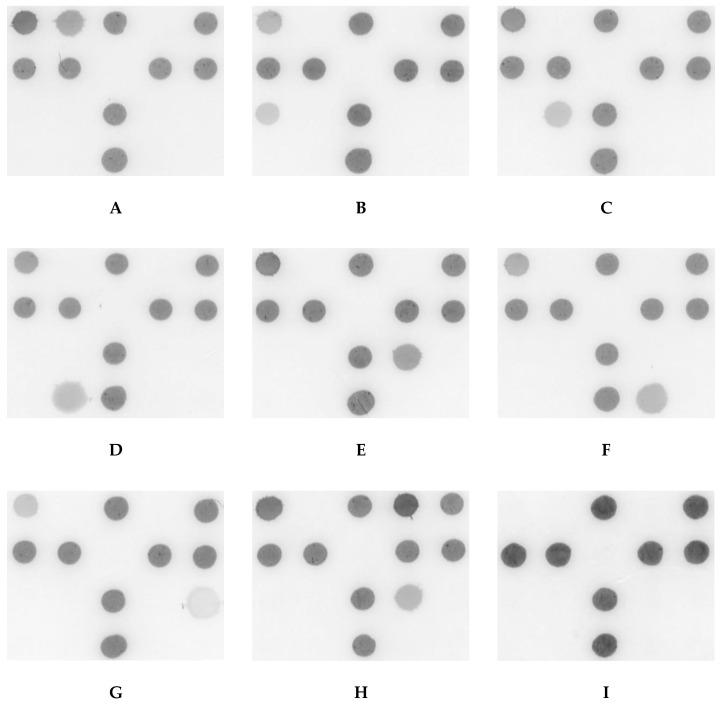
Representative test results of dot hybridization assay for detecting the putative microorganisms of core ocular surface microbiota. (**A**) *Pseudomonas aeruginosa*; (**B**) *Acinetobacter baumannii*; (**C**) *Klebsiella pneumoniae*, (**D**) *Serratia marcescens*, (**E**) oxacillin-sensitive *Staphylococcus aureus*, (**F**) oxacillin-sensitive *Staphylococcus epidermidis*, (**G**) *Propionibacterium acnes*, (**H**) oxacillin-resistant *Staphylococcus aureus*, (**I**) ddH_2_O. The size of each membrane was 1.0 × 0.8 cm.

A DHA model for putative COSM was proposed (Figure 1) and the probes used in the model were shown in Table 1. For obtaining the optimal concentration of each dot, three strains of *P. aeruginosa* (ATCC 27853, BCRC 10944, and BCRC 11864), *Acinetobacter baumannii* (*A. baumannii*) (BCRC 10591, BCRC 15884 and a clinical isolate K043), *Klebsiella pneumoniae* (*K. pneumoniae*) (BCRC 11644, CCUG 15938 and CCUG 26735), *Serratia marcescens* (*S. marcescens*) (BCRC 15326, BCRC 11576 and a clinical isolate K001), *Staphylococcus aureus (S. aureus)* (BCRC 15287, BCRC 14957 and BCRC 15285), *S. epidermidis* (BCRC 10785, and two clinical isolates K049 and K050), and *Propionibacterium acnes* (*P. acnes*) (CCUG 6369, CCUG 4745 and a clinical isolate K007) were used to observe the DHA response. In addition, three isolates of oxacillin-resistant *S. aureus* (K104, K105, K106) were used to obtain the optimal concentration of probe mecA. Each probe′s concentration was determined by serial dilutions of genomic DNA and shown in Table 1 to reach a similar detection limit of approximately 1 pg DNA/μL. The DHA response was demonstrated in Figure 2.

### 2.2. Clinical Subjects

A total of 43 patients, including 22 women and 21 men, participated in this study. The mean age was 66.2 ± 7.4 years (ranged from 47 to 81 years). The mean age of female subjects was 66.8 ± 7 years, while the mean age of male subjects was 65.5 ± 7.8 years. There was no statistical difference in age for male and female subjects receiving cataract surgery. Nineteen subjects received right eye surgery and 24 subjects received left eye surgery.

### 2.3. Blood Culutre with Matrix-Assisted Laser Desorption Ionization-Time of Flight Mass Spectrometry (MOLDI-TOF MS) for Determination of Ocular Surface Microbiota

**Figure 3:**
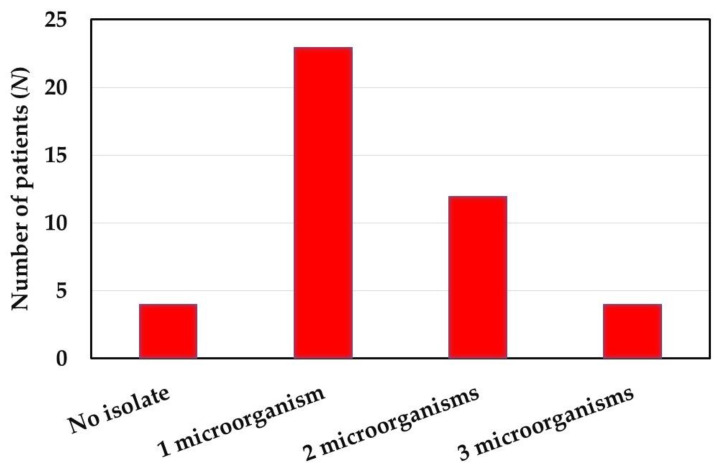
Number of microorganisms isolated from cataract patients preoperatively.

Most subjects (39/43, 90.7%) had positive blood culture and one or more microorganisms were identified by MOLDI-TOF MS. Over half (23/43, 53.5%) of the subjects were isolated with one microorganism. Only four patients had no microorganisms cultured, while four patients had three isolated microorganisms (Figure 3).

A total of 59 microorganisms were isolated from 39 culture-positive subjects, of which most isolates (53/59, 89.8%) were Gram positives. In Table 2, only five strains of *S. aureus* were isolated, whereas nearly half (29/59, 49.2%) of the isolated microorganisms were CoNS. There were only four streptococci, but three strains of *Enterococcus faecalis* were isolated. *Bacillus* species was second only to *Staphylococcus* species in microorganism isolates. There were two Actinobacteria identified, including *Microbacterium aurum* and *Gordonia* spp. In addition, there were only four Gram-negative bacteria identified, including *Morganella* spp., *Serratia marcescens, Citrobacter* spp., and *Brevundimonas* spp. For oxacillin resistance, CoNS was more common than *S. aureus* (14/29 vs. 1/5). Among 15 oxacillin resistant strains, *S. epidermidis* (7/15, 46.7%) was the most common, while only one *S. aureus* strain (1/15, 6.7%) was oxacillin resistant. However, considering the same species, most strains (6/7, 85.7%) of *S. haemolyticus* were oxacillin resistant, and were more common than *S. epidermidis* (7/16, 43.8%). There was only one subject (case no. 41) with oxacillin resistance in both isolates (*S. aureus* and *S. epidermidis*).

### 2.4. DHA Model for Evaluation of Core Ocular Surface Microbiota

**Figure 4:**
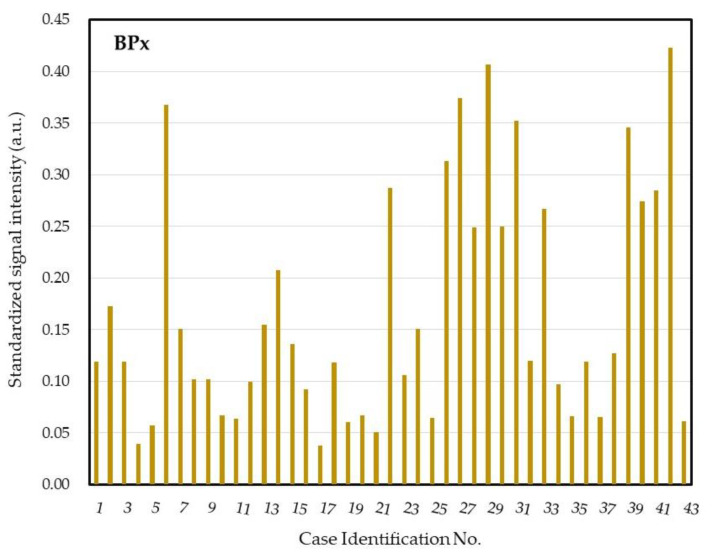
Molecular bioburden of the core ocular surface microbiota of the patients for cataract surgery. a.u. = arbitrary unit.

For patients who underwent cataract surgery, the molecular bioburden of COSM was determined by the dot BPx, a bacteria universal probe (Figure 4). The standardized signal intensities of all dots in the negative control sample (ddH_2_O) were no more than 0.02 arbitrary unit (a.u.), and therefore 0.03 a.u. was assigned as the threshold of each probe to determine a positive reaction. We found diverse bacterial bioburden for these patients, ranging from 0.038 to 0.423 a.u. in the standardized signal intensity, in which 28 (65%) patients had higher bioburden (signal ≥ 0.10 a.u.).

**Figure 5:**
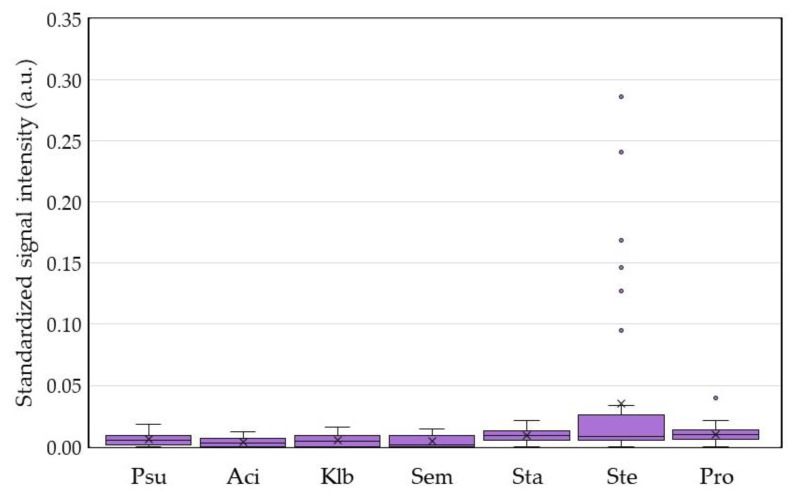
Assessment of target microorganisms in the core ocular surface microbiota dot hybridization model for the patients preparing for cataract surgery. Dots Sta, Ste, and Pro had significantly higher signal intensities than the remaining dots. Dot Ste had a significantly higher signal intensity than dots Sta and Pro. There was no significant signal difference between dots Sta and Pro. Also, there was no significant difference in the signal intensity between any two dots Psu, Aci, Klb, and Sem. a.u. = arbitrary unit.

In regard to the dots for monitoring the microorganisms of COSM, we found no positive detection for Gram-negative pathogens, including *P. aeruginosa*, *A. baumannii*, *K. pneumoniae*, and *S. marcescens* (Figure 5). Although the three dots for Gram-positive bacteria had higher signal intensities than those for detecting Gram-negatives, only seven patients were positive for *S. epidermidis* and one patient was positive for *P. acnes*. This result implied little to no target microorganisms in the defined COSM at the lid margin of these cataract patients.

**Figure 6:**
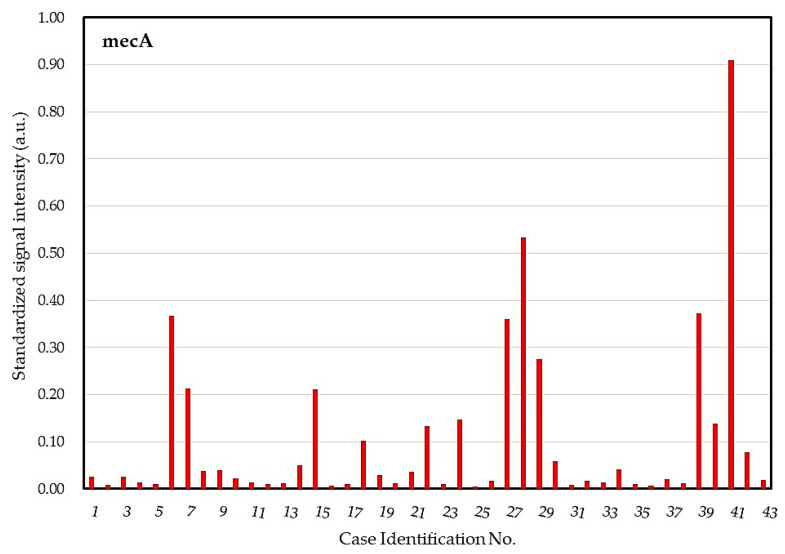
Surveillance of potentially resistant strains in the core ocular surface microbiota dot hybrization model. a.u. = arbitrary unit.

The dot for surveilling potentially resistant strains revealed 20 patients′ microbiota with dot mecA positive (Figure 6), in which 12 patients had a highly positive response (signal ≥ 0.10 a.u.). Different signal intensities of the dot mecA suggested variant levels of microbial bioburden in different subjects or variant numbers of copies of the *mecA* gene in different microbial strains.

### 2.5. Integrative Analysis for COSM from DHA and Blood Culture with MALDI-TOF MS

**Figure 7:**
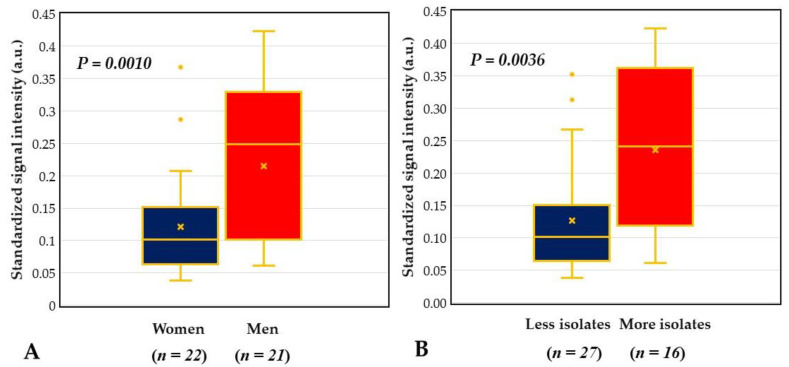
Comparison of bacterial universal probe BPx signal between different groups. (**A**) Men had a significantly higher signal intensity than women. (**B**) The more isolates group had a significantly higher signal intensity than the fewer isolates group; Fewer isolates = patients with none or one isolate; More isolates = patients with two or more isolates. a.u. = arbitrary unit.

However, there was no statistical difference in the number of isolated microorganisms from a culture between women and men, while male patients had a significantly higher mean signal intensity of bacterial universal probe BPx than female patients (0.121 vs. 0.215 a.u., *p* = 0.0147) (Figure 7A). The standardized signal intensity of BPx had no correlation with age but was positively correlated with the number of isolated microorganisms from culture (*p* = 0.48, *p* = 0.0010). We further compared the signal intensity of BPx between the groups with more (patients with two or more isolated microorganisms) and less (patients with none or one isolated microorganism) isolates and found the former had significantly higher signal intensity than the latter (0.236 ± 0.124 vs. 0.127 ± 0.086, *p* = 0.0036) (Figure 7B).

Among the 16 patients with *S. epidermidis* isolated from culture, seven patients were also identified by the probe Ste. However, two patients with probe Ste positive had no *S. epidermidis* isolated by culture. Although DHA could recover two additional strains of *S. epidermidis*, nine strains of *S. epidermidis* were not identified by the probe Ste. The result further supported that the scarcity of microorganisms around the eyelid margin led to the false-negative detection of *S. epidermidis* by the DHA model even when *S. epidermidis* is the most commonly isolated microorganism of the ocular surface microbiota.

**Figure 8:**
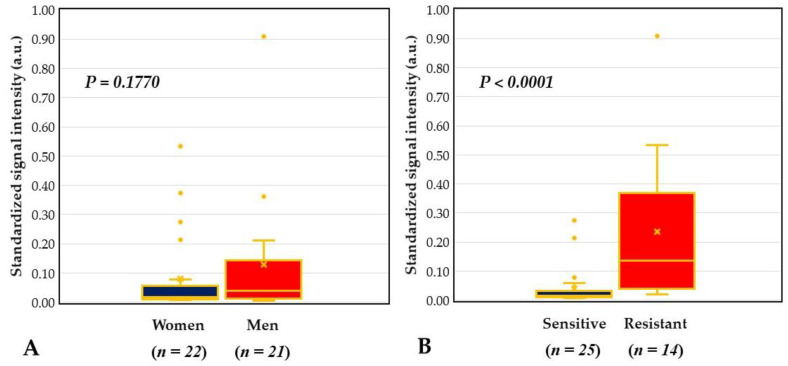
Comparison of antibiotics resistance surveilling probe mecA signal between different groups. (**A**) No significant sex difference in the signal intensity of mecA. (**B**) Patients with oxacillin-resistant strains (resistant group) had higher signal intensity of mecA than those without oxacillin-resistant strains (sensitive group). a.u. = arbitrary unit.

Among the 20 patients with positive *mecA*, there were six patients with all of their isolates susceptible to oxacillin and one patient with no isolated microorganism. Among the 23 patients with negative *mecA*, two patients had oxacillin resistance and both were from the isolated *S. haemolyticus*. Neither age difference was found between patients with oxacillin resistant strains and those without oxacillin resistant strains (*p* = 0.8837), nor correlation was found between patient age and mecA signal intensity (*p* = 0.24, *p* = 0.1183). The male patients had more oxacillin-resistant strains isolated than female patients (10/21 vs. 4/22), but the difference was not statistically significant (*p* = 0.0546). In addition, there was also no significant sex differences in the signal intensity of probe mecA (Figure 8A). However, the patient with the highest signal intensity of mecA (0.909 a.u.) was the male patient with oxacillin resistance in both isolates (*S. aureus* and *S. epidermidis*). In addition, the standardized signal intensity of dot mecA was also positively correlated with the number of isolated microorganisms from blood culture (*p* = 0.44, *p* = 0.0033). Moreover, comparing patients with oxacillin-resistant strains (*n* = 14) from those with oxacillin-sensitive strains (*n* = 25), the signal intensity of mecA of the former was significantly higher than that of the latter (0.235 ± 0.254 vs. 0.038 ± 0.065, *p* < 0.0001) (Figure 8B).

## 3. Discussion

We proposed a DHA model for investigating and monitoring COSM based on the lid margin sample. This DHA model was designed for simultaneous assessment of microbial burden (Figure 4), detection of common pathogens and flora (Figure 5), as well as surveillance of antibiotic resistance (Figure 6) on ocular surface microbiota. We found that the bacterial bioburden was heavier in men and compatible with the number of cultural isolates (Figure 7). In addition, the DHA could detect oxacillin-resistant strains that could not otherwise be isolated by culture (Figure 8). Ocular surface microbiota was found to be changed in the settings of a certain treatment or disease [25,26,27]. Therefore, it can be adopted as a routine clinical practice in the personal risk assessment of ocular surface health, including infection risk of ocular surgery, adverse effect risk of long-term instillation of an eyedrop, intensive treatment risk for severe ocular surface diseases, and so on. Moreover, this model can be modified according to the novel findings of a growing metagenomic survey and the trend of antibiotic susceptibility from the culture of ocular surface microbiota for a specific disease or treatment.

The existence of conjunctival microbiota is still controversial due to its paucibacterial microbiome, which is approximately 150- to 200-fold fewer than the facial skin or the buccal mucosa in terms of bacterial load and may be caused by dynamic washing and antibacterial components of tears [28]. Thus, microbial communities can be transient and difficult to form a stable microbiota over time [6]. Recently, Suzuki et al. found meibum of healthy adult subjects harbors highly diverse microbiota, and the diversity of microbiome decreases with aging and may affect the homeostasis of the ocular surface [29]. The meibum microbiome resembled the conjunctival sac microbiome in young people, whereas the conjunctival sac microbiome became more similar to the eyelid skin microbiome in the elderly. A close relationship of the microbiome was demonstrated among conjunctiva sac, meibum, and eyelid skin. Therefore, the isolated microbiota in the eyelid margin adopted in our study could better represent the ocular surface microbiota by widely collecting microorganisms from these sampling sites.

Both Mshangila et al. [30] and Ratnumnoi et al. [31] found that CoNS was the most common flora of the conjunctiva and lid margin for patients undergoing cataract surgery, which comprised 65.9% and 88.3% of all isolates, respectively. Suto et al. revealed the bacterial isolation rate from conjunctival sac swabbing was about 39.2%, of which CoNS was the most common isolates (57.2%) [32]. However, recently, Matsuura et al. also sampled the ocular surface microbiota by conjunctival sac scraping and approximately two microorganisms (mean 1.99) were isolated from each eye for patients about to undergo cataract surgery [33]. The major microorganisms included *S. aureus*, *S. epidermidis*, CoNS other than *S. epidermidis*, *E. faecalis*, and *Streptococcus* spp. In our study, the mean cultured microorganism from each eye was 1.4 by lid margin swabbing and similarly, the most common isolate was CoNS (*n* = 29, 49.1%), in which *S. epidermidis* (*n* = 16) and *S. haemolyticus* (*n* = 7) were the most commonly isolated CoNS by the MALDI-TOF identification from pediatric blood bottle culture (Table 2). Other isolates identified over three eyes included *S. aureus* (*n* = 5) and *E. faecalis* (*n* = 3). Four *Streptococcus* spp. were isolated and identified to species level, while no single species was recovered over two eyes. The above result was compatible with Matsuura et al. even though the sampling sites were different. However, nine *Bacillus* spp. were isolated and only one isolate (*B. cereus*) could be identified to species level. The identification of *Bacillus* spp. seemed higher than previous reports, suggesting that *Bacillus* spp. may have been a selection effect from different cultures and identification systems or these species were not conjunctival microbiota.

We further compared our study with three similar studies (Table 3) and speculated that the sampling site may influence the number of isolated microorganisms, and the culture system may influence the kinds of isolated microorganisms. The isolated rate of CoNS in our study was less than that of the other studies. Other factors influencing the number of isolated microorganisms for patients who underwent cataract surgery may also include climate, geographic location, urbanization, and so on.

Four eyes had no microorganism isolated by culture, but all eyes had a detectable bacterial signal on the BPx dot of the DHA (Figure 4). Nine subjects with positive *S. epidermidis* culture, five subjects with *S. aureus* culture, and one subject with positive *S. marcescens* culture were not detected by dots Ste, Sta, and Sem. The above result was compatible with paucibacterial microbiome on the ocular surface due to very few microorganisms over the lid margin. For culturable microorganisms, culture is the most sensitive method because only one viable microbe is required for diagnosis. The DHA basically requires target DNA templates from 10 or more microbes in the sample but it has the advantage of detecting nonviable or unculturable microbes. Two eyes with *S. epidermidis* and one eye with *P. acnes* were additionally identified by the dot Ste and Pro, respectively (Figure 5). This result indicated that there was a higher number of nonviable or fastidious microbes over the lid margin of the three eyes.

For patients undergoing cataract surgery, methicillin-resistant CoNS consisted of approximately 12% to 32% of isolated microorganisms, while methicillin-resistant *S. aureus* was about 1.0% to 27.6% [30,32]. In our study, 23.7% (14/59) of the isolates were identified as oxacillin-resistant CoNS and 1.7% (1/59) was oxacillin-resistant *S. aureus* by antimicrobial susceptibility test (Table 2). Oxacillin resistance was more common for strains of CoNS (14/29, 48.3%) than those of *S. aureus* (1/5, 20%), which was compatible with previous studies [30,32]. However, it is worth noting that oxacillin resistance was more prevalent for strains of *S. haemolyticus* (6/7, 85.7%) than those of *S. epidermidis* (7/16, 43.8%).

Chiquet et al. found the *S epidermidis* strains from the endophthalmitis patients harbored higher rates of the *mecA* gene compared to those of the control subjects (54% vs. 11%, *p* < 0.001). The mecA dot in the DHA model played a critical role in monitoring potentially resistant strains on the ocular surface, where almost half of the subjects (20/43, 46.5%) carried the strains with the *mecA* gene (Figure 6). Seven of the *mecA* positive subjects showed oxacillin-sensitive in all of their isolated microorganisms, which suggested mutations in regions of nucleotide repeats within *mecA* in the *Staphylococcus* spp. [34], or perhaps not all microorganisms were recovered from the culture due to the death of some fastidious microorganisms. Strains that contain *mecA* but are phenotypically susceptible can become resistant after antibiotic exposure, which may result in treatment failure if infection occurs [34]. On the contrary, two subjects with oxacillin resistant strains were not *mecA* positive, where the two strains may have carried other resistant genes, such as *mecB*, *mecC*, *mecD*, and other homologs of *mecA* [35]. However, this result suggested the major contributor to oxacillin resistance is *mecA* gene, which is the best candidate for monitoring potentially resistant strains of the ocular surface.

Zhang et al. reported healthy subjects without contact lens wearers had sex differences in the composition of bacterial community based on whole-genome sequencing of the V3 region of the 16S rRNA gene [36]. Wen et al. found that male and female healthy subjects differed in the β diversity of bacterial communities on ocular surface microbiome by using metagenomic shotgun sequencing [37]. In our previous study, we verified the standardized signal intensity (arbitrary units) of the universal bacterial probe (dot BPx) was positively correlated with bacterial bioburden (colony-forming units/mL) in culture [20]. For patients undergoing cataract surgery, we found male subjects had a significantly higher standardized signal intensity of dot BPx than that of the female subjects (Figure 7A). Thus, we concluded that the male subjects had a heavier bacterial bioburden than the female subjects. Moreover, male subjects might have a higher ratio of oxacillin-resistant strains and copies of the mecA gene in their ocular surface microbiome than female subjects (Figure 8A), but this did not reach statistical difference. A more abundant microbial community could protect the ocular surface, which contributes to men with a lower risk of ocular surface disease than women [37], but heavier microbial bioburden may lead to a higher risk of postoperative endophthalmitis in men [38,39,40].

We adopted the pediatric blood bottle system because of its wide-spectrum coverage of aerobic and anaerobic microorganisms. In addition, the sample could be equally distributed for the culture and the DHA. However, multiple culture media was not adopted in this study because there was a limited number of microorganisms on the ocular surface. In addition, some fastidious microorganisms may not be cultured. Moreover, microorganisms may have various growth rates in different culture systems, in which certain microorganisms may be selected by a specific culture system. Thus, discrepant results are would inevitably occur between any two different culture systems as well as between a culture system and the DHA assessment.

*P. acnes* (now renamed as *Cutibacterium acnes*), a common microorganism recognized in the studies of ocular surface flora and microbiome [29,41,42,43], was not identified by the pediatric blood bottle culture and MALDT-TOF procedure. This result may be due to microbial selection during the culture procedure, of which an additional sample for anaerobic blood bottle culture could have a higher opportunity to recover this aerotolerant anaerobic bacterium. In addition, topical instillation of 4% Sulfamethoxazole for one week preoperatively may also greatly reduce the number of *P. acnes* over the lid margin and led to lower signal intensities of the dot Pro for these subjects (Figure 5). Observing the very low signal intensities of the dots for all target microorganisms, the result was compatible with low risk of infection for these patients, who had no occurrence of endophthalmitis after cataract surgery.

## 4. Materials and Methods

### 4.1. Subjects

This was a prospective cohort study, enrolling patients for cataract surgery in Kaohsiung Chang Gung Memorial Hospital (CGMH) between 1 November 2019 and 31 January 2020. Informed consent was obtained from all subjects, and all procedures adhered to the Declaration of Helsinki on human subjects. Institutional Review Board/Ethics Committee approval (code no. 201600708B0) was obtained from the Committee of Medical Ethics and Human Experiments of CGMH, Taiwan. To decrease ocular surface bioburden and the risk of postoperative endophthalmitis, each patient routinely received binocular instillation of 4% sulfamethoxazole four times per day for one week before operation. Participants who were aged less than 45 years, had acute ocular or eyelid inflammation, topically applied topical or systemic antibiotics (except topical 4% sulfamethoxazole preoperatively), wore contact lens within 3 months, and underwent ocular or eyelid surgery within 3 months were excluded.

### 4.2. Sample Collection

Before the disinfection procedure of cataract surgery, two sterile 6” cotton-tipped applicators (cotton tip 0.8 × 2.0 cm, Team Power Healthcare Ltd., New Taipei, Taiwan) were used to collect the sample of eyelid margin. The first applicator was used to swab upper and lower eyelid margin with gentle pressure, and the second applicator was used to provide a counterforce to facilitate the sampling of the first applicator. After sampling, the first applicator was inserted into a 1.5 mL sterile microcentrifuge tube containing 1 mL of ddH_2_O and then broken off into two parts: the anterior part with cotton was shorter than the inner depth of the tube in order to fit inside the tube under a sterile procedure. The tube was then kept at 4 °C within three days before DNA extraction. Before DNA extraction, the broken cotton-tipped applicator was discarded after the cotton tip was pressed on the inner wall of microcentrifuge tube for draining fluid back to the tube as much as possible under sterile procedure. After vortex, 150 µL fluid sample was aspirated and injected into the pediatric blood bottle (BD BACTEC PEDS Plus/F, US) for microbial culture. The remaining fluid in the microcentrifuge tube was centrifuged at 13,000× *g* for 10 min. The precipitate was resuspended in 100 µL phosphate-buffered saline and used for microbial DNA extraction. The extracted DNA was stored in the −70 °C refrigerator for core microbiota assessment.

### 4.3. Identification of Cultured Microbiota by the MOLDI-TOF Mass Spectrometry

After the automatic blood culture system (BACTEC™ FX; BD Diagnostics Systems, Sparks, MD, USA) had alarmed for positive microbial bottles, the microorganism in the pediatric blood bottle was subcultured on solid media, including blood agar, EMB agar, and chocolate agar. After 24 h of incubation at 35 °C with 5% CO_2_, all of the isolated colonies were identified by MALDI-TOF technique using the MALDI Biotyper system (Bruker Daltonics, Bremen, Germany). After that, all of the isolated colonies were tested by disc diffusion method with Mueller–Hinton agar and were automatically interpreted according to CLSI guidelines by Adagio™ Antimicrobial Susceptibility Testing System.

#### 4.3.1. Sample Preparation for MALDI-TOF MS Analysis

The colonies of microorganisms obtained from subcultures were applied as thin films onto a 96-spot steel target (Bruker Daltonics, Bremen, Germany) and then overlaid with 1 μL of 70% (*v/v*) aqueous formic acid, followed by air drying. Next, 1 μL of MALDI matrix (α-cyano-4-hydroxycinnamic acid in 50% acetonitrile and 2.5% trifluoroacetic acid) was applied to the colony and dried naturally before testing. A Bacterial Test Standard (Bruker Daltonics) was included for calibrating the instrument and validating the operation. Each sample was spotted at least three times on the plate.

#### 4.3.2. Identification of Ocular Surface Microobiota by MALDI Biotyper

Mass spectra (2 to 20 kDa) were obtained automatically, using the Autoflex III MALDI-TOF MS equipped with a nitrogen laser, working in positive linear mode and controlled by the custom-made software FlexControl. Spectra were then analyzed via the Flex Analysis software (Bruker Daltonics, Bremen, Germany). The replicates with intensity <104 a.u. and those with a profile significantly different from the others were excluded. The MALDI BioTyper software, version 3.1 and library (5989 isolates; Bruker Daltonics, Bremen, Germany) were adopted to identify the microbial strains. The automated workflow allowed standardized sample acquisition (accumulating 300 to 500 shots of high quality from different spot positions), data processing, and final identification. The standard Bruker explanatory criteria were adopted, including unreliable identification (score 0.000 to 1.699), probable genus identification (score 1.700 to 1.999), secure genus and probable species identification (score 2.000 to 2.299), and highly probable species identification (score 2.300 to 3.000).

### 4.4. DNA Extraction and PCR Amplification

The microbial DNA in the precipitate was extracted using a commercial kit (DNA Micro kit Qiagen, Germany), and then stored at −70 °C. Bacteria universal primers 2F (5′-digoxigenin-TTGTACACACCGCCCGTC-3′) and 10R (5′-digoxigenin- TTCGCCTTTCCCTCACGGTA-3′) were used to amplify a DNA fragment that encompassed a portion of the 16S rRNA gene, the 16S-23S rRNA spacer region, and a portion of the 23S rRNA gene [22]. The antibiotics-resistant gene mecA was amplified using primer pairs forward (mecF, 5′-digoxigenin- AAAATCGATGGTAAAGGTTGGC-3′) and reverse (mecR, 5′-digoxigenin- AGTTCTGCAGTACCGGATTTGC-3′) [44]. Each primer was labeled with a digoxigenin molecule at its 5′ end. The PCR reaction mixture (25 μL) consisted of 2.5 μL template DNA, 0.2 μM each primer, and other necessary reagents obtained from the PCR kit (JMR-THS5, JMR Holdings, Inc., St. Augustine, FL, USA). Cycling conditions of multiplex PCR were as follows: initial denaturation (95 °C, 5 min); 35 cycles of denaturation (95 °C, 30 s), annealing (55 °C, 45 s), and extension (72 °C, 45 s); followed by a final extension at 72 °C for 7 min. Negative control was performed with each run by replacing the template DNA with sterile water.

### 4.5. The Dot Hybridization Assay for Assessing COSM

In this specially-designed DHA (Figure 1, Table 1) for assessing COSM, there were one bacteria universal dot (BPx; designed from conserved sequences at the 3′ end of the 16S rRNA gene) for estimating overall bacterial bioburden, seven bacteria-specific dots (four hazardous Gram-negative group: Psu, Aci, Klb, and Sem; three Gram-positive group: Sta, Ste, and Pro; all designed from the 16S-23S rRNA internal transcribed spacer) for detecting bacteria of putative core ocular surface microbiota, and one dot for surveilling antibiotic resistance of ocular surface bacteria (mecA).

#### 4.5.1. Immobilization of Oligonucleotide Probes on a Nylon Membrane

The procedure of oligonucleotide probes secured on a nylon membrane was described in a previous study [45]. In brief, each probe was diluted 1:1 (final concentration, 10 µM) with a tracking dye solution and spotted on a nylon membrane with positive charge (Roche, Mannheim, Germany) using a bio-spotter (SR-A300; EZlife Technology, Taipei, Taiwan). For specifying the loci of these probes and providing the reference of probe signal standardization, a digoxigenin-labeled irrelevant probe (dot M) was used as a marker and spotted on the membrane (1.0 × 0.8 cm; Figure 1). After all probes had been spotted, the membrane was exposed to a shortwave UV (Stratalinker 1800; Stratagene, La Jolla, CA, USA) for 30 s to immobilize the probes on this membrane.

#### 4.5.2. Detection of Microbial DNA with the COSM DHA

A 10-µL aliquot of the PCR product was used for the DHA assessment. The procedures for prehybridization and hybridization (55 °C for 60 min) have been described previously [43]. The reagents used in this study included alkaline phosphatase-conjugated anti-digoxigenin antibodies (Anti-Digoxigenin-AP, Fab fragments, Roche) and color development using phosphatase substrates (NBT/BCIP Stock Solution, Roche). The images of hybridized arrays were captured with a scanner (Perfection V600 Photo; Epson, Tokyo, Japan), where the hybridized dots (400 µm) could be read by naked eyes.

#### 4.5.3. Quantification of the Signals for Each Dot in the DHA

The intensity of hybridization signal was quantified using ImageJ (National Institutes of Health, Bethesda, MD, USA) and described previously [19,20]. In brief, each captured image was adjusted to a fixed size (500 × 400 pixels) and transformed to greyscale. The grey level of each hybridized dot was then detected and recorded. Grey levels of the image background were determined by averaging the negative controls (NC; tracing dye only). The mean grey levels of markers were measured by averaging the levels of all marker dots (dot M). The corrected intensity of markers was obtained by subtracting the background level from the mean grey level of markers. The corrected intensity of a dot was obtained by subtracting the background level from the grey level of a dot. The standardized intensity of a dot was defined as the corrected intensity of a dot divided by the corrected intensity of markers in the same image.

### 4.6. Data Analysis

The Mann–Whitney U test was used to compare the standardized signal intensities of each dots in the DHA between groups, including sex, less and more isolates groups, oxacillin sensitive and resistant groups for the patients undergoing cataract surgery. Fisher exact test was adopted to analyze the significance between sex and oxacillin susceptibility. Spearman′s rank correlation coefficient was used to explore the association between age, number of isolates, and signal intensities of each dot in the DHA. GraphPad Prism v.8.4.3 for Windows (GraphPad Software, San Diego, CA, USA) was used for statistical analysis. A *p*-value of <0.05 was considered statistically significant.

## 5. Conclusions

The newly proposed DHA model for investigating and monitoring COSM seems to be clinically adoptable, especially for patients undergoing cataract surgery, since it enables assessment of bacterial bioburden, detection of target pathogens and microorganisms, and surveillance of antibiotic resistance in the ocular surface microbiota. Bacterial bioburden shown by the bacterial universal dot was heavier in men and was associated with the number of bacterial isolates, which was compatible with a higher risk of endophthalmitis in male patients who underwent cataract surgery. Moreover, no occurrence of endophthalmitis for these patients was compatible with low signal intensities of pathogen-specific probes. Furthermore, the mecA dot could detect the oxacillin-resistant strains, which could not be isolated by culture. This model can be modified to assess or monitor the change of ocular surface microbiota for a specific disease or different treatments according to the novel findings of growing metagenomic surveys and the trend of antibiotic susceptibility from culture.

## Figures and Tables

**Table 1:** Oligonucleotide Probes Used in the Core Ocular Surface Microbiota Dot Hybridization Assay.

Dot	Assessment Target	Probe Sequence(s) (5′–3′)	Conc. (μM)	Ref.
BPx	All bacteria	GGGGCTAAGTCGTAACAAGGTAGCCGTAtttttttttt ^b^	20	[19,20]
Psu ^a^	*Pseudomonas aeruginosa*	GTTCTTTAAAAATTTGGGTATGTGATAGAA CAGTGACCAGATTGCTTGGGGTTATATtttttttt ^b^	10 10	[19,20]
Aci	*Acinetobacter* *baumannii*	CGGTAATTAGTGTGATCTGACGAtttttttttt ^b^	10	[23]
Klb	*Klebsiella pneumoniae*	CTTAAAGAACCTGCCTTTGTAGTGCTC	20	[19,20]
Sem ^a^	*Serratia* *marcescens*	AAGGTACTGCGCGTGACTGTATGGtttttttttt ^b,c^; CATATAGTCCGGTATTTAATACTTCAGAGTtttttttttt ^b,c^	20 20	-
Sta	*Staphylococcus aureus*	CGTTATTCCGCATCTTCTGAAGAAGAttttt	20	[22]
Ste	*Staphylococcus epidermidis*	TTGAATAACAATTCAAAATATGGTGGAttttttttttt ^b^	20	[22]
Pro ^a^	*Propionibacterium acnes*	TTGCTGTATGTGTTCGTGCGACtttttttttt ^b^; GAGCATCTTATTTTTTGTGTGGCTTGTGttttttttttttttt ^b^	20 20	[24]
mecA	*Potentially resistant* strain	TGATGGTATGCAACAAGTCG	10	[22]
M	Marker	5′-digoxigenin-TCCTCCGCTTATTGATATGC	10	[19,20]

^a^ A dot with mixed probes. ^b^ Multiple bases of thymine (t) were added to the 3′ end of the probe. ^c^ A newly developed probe.

**Table 2:** Different Microorganisms Isolated from Eyelid Margin of Cataract Patients Preoperatively.

Isolated Microorganisms	Number of Isolates	Number of Oxacillin Resistant Strains
**Gram Positive Microorganisms**		
*Staphylococcus aureus*	5	1
*Staphylococcus epidermidis*	16	7
*Staphylococcus haemolyticus*	7	6
*Staphylococcus warneri*	2	0
*Staphylococcus sciuri*	1	1
Unspecified CoNS	3	0
*Streptococcus mitis*	1	0
*Streptococcus oralis*	2	0
*Streptococcus salivarius*	1	0
*Enterococcus faecalis*	3	0
*Bacillus cereus*	1	0
*Bacillus* spp.	8	0
*Paenibacillus* spp.	1	0
Unspecified Gram-positive bacilli	2	0
**Gram Negative Microorganisms**		
*Microbacterium aurum*	1	0
*Gordonia* spp.	1	0
*Morganella* spp.	1	0
*Serratia marcescens*	1	0
*Citrobacter* spp.	1	0
*Brevundimonas* spp.	1	0

CoNS = coagulase negative staphylococci.

**Table 3:** Comparison of the Isolated Number of Cultured CoNS on Ocular Surface in Similar Research Subjects.

Research	Sampling Sites	Culture System	No. (%) of CoNS	No. (%) of *S. epidermidis*	Ref.
Mshangila et al. (Uganda, *n* = 131)	Lower eyelid margin and inferior conjunctival sac	Brain–heart infusion broth	91/138 (65.9%)	70/138 (50.7%)	[31]
Ratnumnoi et al. (Thailand, *n* = 120)	Eyelid margin and conjunctiva	Blood agar and chocolate agar	106/115 (92.2%)	N.A.	[32]
Suto et al. (Japan, *n* = 579)	Inferior conjunctiva sac	Blood agar and chocolate agar	164/284 (57.7%)	164/284 (57.7%)	[33]
In this study (Taiwan, *n* = 43)	Upper and lower lid margin	Pediatric blood bottle	29/59 (49.2%)	16/59 (27.1%)	

CoNS = Coagulase Negative Staphylococci. N.A. = not available.

## Abbreviations

a.u.	Arbitrary Unit
CGMH	Chang Gung Memorial Hospital
CoNS	Coagulase Negative Staphylococci
COSM	Core Ocular Surface Microbiota
DHA	Dot Hybridization Assay
MOLDI-TOF	Matrix-Assisted Laser Desorption Ionization-Time Of Flight
MS	Mass Spectrometry
